# Influence of Adhesive Systems on the Mechanical and Physical Properties of Flax Fiber Reinforced Beech Plywood

**DOI:** 10.3390/polym13183086

**Published:** 2021-09-13

**Authors:** Johannes Jorda, Günther Kain, Marius-Catalin Barbu, Alexander Petutschnigg, Pavel Král

**Affiliations:** 1 Forest Products Technology and Timber Construction Department, Salzburg University of Applied Sciences, Markt 136a, 5431 Kuchl, Austria; jjorda.lba@fh-salzburg.ac.at (J.J.); marius.barbu@fh-salzburg.ac.at (M.-C.B.); alexander.petutschnigg@fh-salzburg.ac.at (A.P.); 2 Department for Furniture and Interior Design, Higher Technical College Hallstatt, Lahnstraße 69, 4830 Hallstatt, Austria; 3 Faculty for Furniture Design and Wood Engineering, Transilvania University of Brasov, B-dul. Eroilor nr. 29, 500036 Brasov, Romania; 4 Department of Wood Science and Technology, Mendel University, Zemědělská 3, 61300 Brno, Czech Republic; kral@mendelu.cz

**Keywords:** wood-based composite, fiber reinforced plywood, flax fiber

## Abstract

In order to improve the acceptance of broader industrial application of flax fiber reinforced beech (*Fagus sylvatica* L.) plywood, five different industrial applicated adhesive systems were tested. Epoxy resin, urea-formaldehyde, melamine-urea formaldehyde, isocyanate MDI prepolymer, and polyurethane displayed a divergent picture in improving the mechanical properties—modulus of elasticity, modulus of rupture, tensile strength, shear strength and screw withdrawal resistance—of flax fiber-reinforced plywood. Epoxy resin is well suited for flax fiber reinforcement, whereas urea-formaldehyde, melamine urea-formaldehyde, and isocyanate prepolymer improved modulus of elasticity, modulus of rupture, shear strength, and screw withdrawal resistance, but lowered tensile strength. Polyurethane lowered the mechanical properties of flax fiber reinforced plywood. Flax fiber reinforced epoxy resin bonded plywood exceeded glass fiber reinforced plywood in terms of shear strength, modulus of elasticity, and modulus of rupture.

## 1. Introduction

Wood is a natural, polymeric, cellular fiber composite that is broadly available and has been used for all kinds of application purposes throughout the history of mankind [1]. To overcome solid wood disadvantages of anisotropy, biodegradability, and dimensional limitations, respectively, various wood-based products such as cross laminated timber (CLT), plywood, oriented strand board (OSB), particleboard (PB), or medium/high density fiberboard (MDF/HDF) have been developed. Natural caused solid wood inhomogeneity are thereby reduced by downsizing raw material geometry [2] and creating homogeneous composite material products with the support of joining materials [3].

Plywood is considered to be the oldest wood-based composite material based on a laminar structure with two distinct fields of application for structural construction purposes and furniture/interior design products [3], and also for several applications for niche market products like transportation, construction, sport equipment, etc. [4]. To enhance the mechanical properties of wood-based products such as plywood and laminated veneer lumber (LVL), fiber reinforcement is well discussed and several experimental studies have been conducted, primarily focusing on synthetic glass and carbon fiber reinforcement, dating back to the 1960s [5]. 

Bal et al. (2015) reinforced phenol-formaldehyde (PF) bonded poplar (Samsun I-77/51 clone) plywood with woven glass fiber (GF) fabric, significantly improving the modulus of elasticity and modulus of rupture for perpendicular samples, and noted a decreasing factor for inequalities between parallel and perpendicular specimens. In addition, density increased whereas thickness swelling and water absorption decreased [6]. Furthermore, screw withdrawal resistance, screw-head pull-through, and lateral nail resistance of glass-fiber reinforced phenol-formaldehyde bonded plywood improved significantly aside from increasing maximum load capacity [7]. Liu et al. (2019) conducted research on different experimental plies of poplar (Populus euramenicana), eucalyptus (Eucalyptus grandis), poplar/eucalyptus, and carbon fiber reinforced plywood for construction formwork [8]. Veneers were bonded with PF resin, whereas carbon fiber fabric was impregnated with epoxy resin and used for bonding carbon fiber to veneer. The combination of different wood species improved flexural plywood performance, which was surpassed by carbon fiber reinforcement. The position of the fiber reinforcement over the plywood cross section is significant for its performance. Surface fiber reinforcement increases the longitudinal modulus of elasticity and the modulus of rupture. In addition, it improves the ultimate load carrying capacity of plywood and influences the failure mode to shear delamination failure caused by the strengthened surface layer. Auriga et al. (2020) studied the effect of randomly unidirectional parallel and perpendicular orientated carbon fiber (CF) reinforcement located internal of the melamine-formaldehyde (MUF) glue line of veneer plies. CF reinforcement was located at two different positions: external at the outer glue line and internal surrounding the core veneer ply. The results displayed increasing modulus of rupture (MOR) and modulus of elasticity (MOE) and the influence of fiber reinforcement location on MOR and MOE [9]. Guan et al. (2020) evaluated the three point bending performance of unidirectional CF (EL 203631 N/mm^2^) reinforced eucalyptus (EL 11619 N/mm^2^)/poplar (EL 6751 N/mm^2^) epoxy resin bonded plywood (thickness 17.5 mm, 17.8 mm, 17.65 mm, 18.0 mm) by digital image correlation (DIC) and finite element analysis (FEA), concluding the usability of FEA for the prediction of material failure behavior [10].

The studies display the effectivity of fiber reinforcement in order to improve physical and mechanical properties of laminar structured wood-based products. Due to rising consumer awareness and resource scarcity, fiber reinforcement based on natural fibers such as flax, hemp, ramie, or basalt can be used in this multilayer laminar composite structure to overcome negative impacts on environmental and resource availability issues. While the concept of natural fiber reinforcement such as flax is not new, research efforts of the last decades have been dominated by synthetic based fiber reinforcement [11].

Several studies have been conducted to investigate the influence of different natural non-/lignocellulose-based fibers in improving the mechanical and physical properties of solid wood and wood-based products. Speranzini and Tralascia (2010) reinforced LVL and solid wood with (FRP) fiber reinforced plastic glass and carbon fibers and natural fibers such as basalt, flax, and hemp. The four point bending test revealed a lower MOR and MOE for natural fibers compared to FRP reinforcement, but still significant improvements compared to the non-reinforced samples [12]. Moezzipour et al. (2017) studied the effect of kenaf and date palm fiber reinforcement on the mechanical and physical properties of horn beam plywood bonded with urea-formaldehyde (UF). Concluding the effectivity of utilizing natural fibers for reinforced plywood products to enhance mechanical performance [13]. Kramár et al. (2020) used non-/impregnated basalt scrim with an area weight of 360 g/m^2^ to enhance the mechanical properties of MUF bonded PB. The effects of different fiber reinforcement positions within the structure on MOR, MOE, internal bond (IB) strength, screw withdrawal resistance (SWR), and thickness swelling (TS) were examined. The study revealed that basalt fiber scrim located at the outer positions significantly improved the strength-to-weight-ratio of particleboards [14]. Jorda et al. (2020) investigated the influence of flax-fiber-reinforcement bonded with epoxy-resin on three-dimensional molded plywood. Improved load capacity and stiffness of flax-fiber reinforced molded plywood structures could be measured [15]. Valdes et al. (2020) reinforced CLT with flax fiber fabrics bonded with bicomponent thixotropic epoxy resin [16]. The study showed that reinforcement of three-layered solid wood panels (SWP) significantly improved load-carrying capacity and stiffness, while the effect for five-layered panels was negligible. 

Concluding from the studies, epoxy resin is the main source for bonding fiber reinforced wood-based composites with synthetic and natural fibers. Some attempts had been made to use PF, UF, and MUF [6,7,8,9,13,14]. For broader industrial production applications, the use of different adhesives is desirable due to the high production costs of epoxy resin systems. Based on its lignocellulosic origin, flax fiber may be bonded with standard industrial plywood adhesives to improve mechanical properties and contribute to broader industrial applications due to limited production process changes and investment costs.

The aim of this study was to investigate the influence of standard industrial adhesives such as UF, MUF, polyurethane (PU-K), isocyanate MDI based pre-polymer (PU-AN), and epoxy resin on the mechanical properties (modulus of elasticity, modulus of rupture, bending strength, tensile strength shear strength, screw withdrawal resistance) of woven flax fiber fabric reinforced beech (*Fagus sylvatica* L.) plywood. The effect of resin type and fiber reinforcement on the panel characteristics named before were evaluated using multivariate statistics.

## 2. Materials and Methods

### 2.1. Materials and Sample Preparation

Pre-conditioned (20 °C, 65% relative air humidity) zero defect rotary cut beech (*Fagus sylvatica* L.) veneers (distributed by Europlac, Topolčany, Slovakia) with the dimensions of 0.75 m × 0.75 m, a thickness of 2.2 mm, an average density of 0.72 g/cm³, and an average moisture content of 12% were used in this study as wooden raw material.

**Figure 1:**
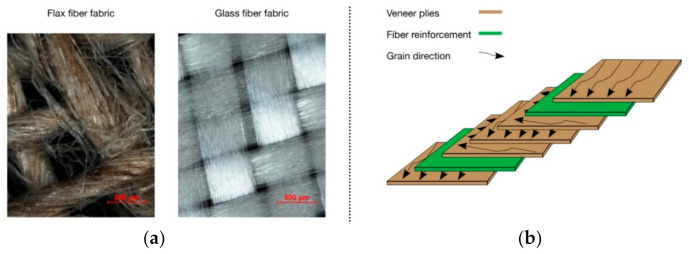
(**a**) Flax fiber vs. glass fiber fabric (**b**) Lay up of 5 × 90° reinforced plywood.

Twill woven flax fabric LINEO FlaxPly Balanced Fabric 200 (Ecotechnilin, Valliquerville, France) with a thickness of 0.4 mm, a density of 1.27 g/cm^3^, and a grammage of 200 g/m^2^ acted as fiber reinforcement. For epoxy resin synthetic fiber reinforcement, textile reference samples of a twill woven e-glass fabric (distributed by DD Composite, Bad Liebenwerda, Germany) with a thickness of 0.5 mm and a grammage of 200 g/m^2^ was used (Figure 1a). 

Five different commercially available and industrially applied adhesives were used to bond the veneer plies and the flax and glass fiber fabric. Epoxy resin SR GreenPoxy 56 (Sicomin, Chateneuf les Matigues, France) with hardener SD 7561 was used. Specifications were: density of 1.198 g/cm^3^ and 0.971 g/cm^3^, respectively, initial viscosity of 0.7 Pa*s and a resin/hardener ratio of 100:36 g were used (press time 13 h, temperature 20 °C). The adhesive application per glue line was set to 200 g/m^2^. Polyurethane adhesive (PUR) Polyurethan 501 Kleiberit (Kleiberit, Weingarten, Germany) with a density of 1.13 g/cm^3^ and viscosity of 7.50 Pa*s (press time 1 h, temperature 20 °C, pressure 0.6 N/mm^2^). The adhesive application per glue line was set to 150 g/m^2^. Isocyanate MDI based prepolymer adhesive PUR system 2010 AkzoNobel (Akzo Nobel, Stockholm, Sweden) (PU-AN) was used with a density of 1.160 g/cm^3^ and viscosity of 6.0 to 19.0 Pa*s (press time 22 min, temperature 20 °C, pressure 2 N/mm^2^). Adhesive application per glue line was set to 200 g/m^2^. Urea-formaldehyde (UF) 1274 Akzo Nobel (Akzo Nobel, Stockholm, Sweden) with hardener 2545 Akzo Nobel with a density of 1.300 g/cm^3^ and 1.450 g/cm^3^, respectively, was used with a viscosity of 1.5 to 3.5 Pa*s/2.0 to 10.0 Pa*s and a resin/hardener ratio 100:20 g (press time 10 min, temperature 90 °C, pressure 1.8 N/mm^2^). Glue amount per glue line was set to 160 g/m^2^. Melamine urea-formaldehyde (MUF) 1247 Akzo Nobel (Akzo Nobel, Stockholm, Sweden) was also used with hardener 2526 Akzo Nobel, a density of 1.270 g/cm^3^, respectively, 1.070 g/cm^3^, a viscosity of 10 to 25 Pa*s/1.7 to 2.7 Pa*s, and a resin/hardener ratio 100:50 g (press time 12 min, temperature 65 °C, pressure 2 N/mm^2^). Glue amount per glue line was set to 300 g/m^2^.

Two lay-ups of plywood were introduced (Figure 1b). The reference samples consisted of five 90° cross laid veneers layers, whereas the fiber reinforced samples consisted of the identical five 90° cross laid veneers layers with two layers of flax or glass fabric (Figure 1b), respectively. These were located in the first glue line on each side in order to improve tensile strength under bending and to minimize the effect of shear stress.

Based on the lay-up, boards with dimensions of 600 mm × 600 mm and a thickness of 10 mm for the non-fiber reinforced reference samples and respectively 11.2 mm for the flax and glass fiber reinforced were produced using a Höfler HLOP 280 press (Taiskirchen, Austria). Lay-up and adhesive application were carried out manually. Adhesive application was controlled by weighing with a KERN ITB 35K1IP device (Baligen-Frommern, Austria). The boards were pressed according to the specific parameters given for each singular adhesive type. Before further testing, the boards where stored for conditioning until mass constancy under constant climate conditions (relative humidity 65%, 20 °C) was achieved. Test specimens were cut from these boards for density, moisture content (MC), tensile- (TS), shear strength (SS), and screw withdrawal resistance (SWR) (Table 1).

### 2.2. Testing

**Figure 2:**
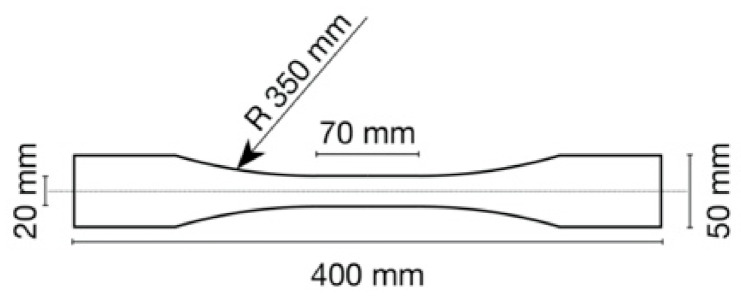
Tensile strength specimen dimensions.

The density was determined according to EN 323:2005 [17], the moisture content according to EN 322:2005 [18] with the specimen size 50 mm × 50 mm, and obtained from bending test specimens after testing. The tensile strength (TS) was measured according to DIN 52377 [19] with specimen dimensions given in Figure 2.

**Figure 3:**
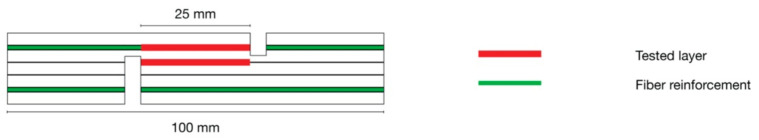
Shear test specimen made of five veneer plies both sides fiber reinforced.

The shear strength (SS) was determined based on EN 314:2005 [20,21] with specimen dimensions of 100 mm × 25 mm (Figure 3).

Modulus of rupture (MOR) and modulus of elasticity (MOE) were determined by a three-point bending test according to EN 310:2005 [22] with specimen dimensions of 250 mm × 50 mm for reference samples and 274 mm × 50 mm for fiber reinforced samples. The screw withdrawal resistance (SWR) was measured according to EN 320:2011 [23] with specimen dimensions of 50 mm × 50 mm and thread screws of ST 4.2 mm. TS, SS, MOR, MOE, and SWR was determined using a Zwick/Roell 250 8497.04.00 test device (Zwick/Roell, Ulm, Germany) and constant climatic conditions (relative humidity 65%, ambient temperature 20 °C). For the statistical analysis, IBM SPSS was used for the descriptive statistics, correlation, and two-way ANOVAs with the consideration of the first order interaction effects for determining the influence of the factors “type of fiber reinforcement” and “adhesive type”.

## 3. Results and Discussion

### 3.1. Density

The results (Table 2) displayed a low effect Pearson correlation between density and applied amount of glue (*p*-value 0.01; R^2^ = 0.065) as well as the significant influence of the factors “type of adhesive” (*p*-value 0.00) and the influence of “fiber reinforcement” (*p*-value 0.00).

Epoxy resin bonded flax fiber reinforced samples with a mean density of 0.843 (standard deviation (SD) = 0.009) g/cm^3^ increased up to 5.5%, respectively 8.8% for glass fiber reinforcement with a mean density of 0.870 (SD = 0.015) g/cm^3^ compared to the reference with a mean density of 0.799 (SD = 0.007) g/cm^3^. The urea-formaldehyde flax reinforcement with a mean density of 0.808 (SD = 0.013) g/cm^3^ increased up to 4.1% compared to the reference mean density of 0.776 g/cm^3^. The melamine urea-formaldehyde bonded flax reinforced sample mean density 0.844 (SD = 0.012) g/cm^3^ increased by 7.0% compared to the reference mean density of 0.789 (SD = 0.012) g/cm^3^. The isocyanate MDI based prepolymer adhesive (PU-AN) flax reinforcement mean density 0.816 (SD = 0.009) g/cm^3^ increased by 6.7% compared to the reference mean density with 0.765 (SD = 0.008) g/cm^3^. PUR bonded flax fiber reinforcement increased by 0.5% with a mean density of 0.794 (SD = 0.014) g/cm^3^ in comparison to the reference mean density of 0.790 (SD = 0.012) g/cm^3^. 

The mean density of flax fiber reinforcement increased between 0.032 g/cm^3^ (4.1%) and 0.055 g/cm^3^ (7.0%) with the exception of PUR with 0.004 g/cm^3^ (0.5%). Enhanced mean density for the fiber reinforcement specimen can be explained by the additional amount of adhesive for the supplementary glue lines and the layers of woven fiber fabric. In addition, density of the different test groups was influenced by the specific adhesive density. The resin density range was 1.13 g/cm^3^ to 1.3 g/cm^3^. In detail, the density for epoxy resin was 1.198 g/cm^3^, UF 1.3 g/cm^3^, MUF 1.270 g/cm^3^, PU-AN 1.16 g/cm^3^, and PUR 1.130 g/cm^3^.

According to Wagenführ and Scholz (2008), density is one of the main influencing parameters for plywood properties, besides the veneer thickness and the solid resin content. Increasing board density correlates with increasing compression strength, enhancing MOE and TS [24].

The low standard deviation values for each test group indicates an even glue application and plywood board production process.

### 3.2. Moisture Content

Epoxy resin bonded flax fiber reinforced MC mean of 9.46 (SD = 0.14)% decreased by 3.76%, respectively 18.31% for the fiber reinforced specimen, compared to the reference mean of 9.83 (SD = 0.14)%. UF bonded flax fiber reinforced MC mean of 10.27 (SD = 0.14)% increased slightly by 0.58% in contrast to the reference mean of 10.27 (SD = 0.09)%. MUF bonded flax fiber reinforced MC mean of 11.42 (SD = 0.04)% decreased by 3.63% compared to the reference MC mean of 11.85 (SD = 2.00)%. Comparability is to be questioned by the standard deviation of SD = 2.00. Isocyanate MDI based prepolymer adhesive (PU-AN) flax fiber reinforced MC mean of 9.75 (SD = 0.32)% decreased by 6.7% in contrast to the reference MC mean of 10.45 (SD = 0.15)%. The PUR bonded flax fiber reinforced MC mean of 8.84 (SD = 0.04)% decreased by 15% compared to the reference MC mean of 10.04 (SD = 0.05)% (Table 3).

It was concluded that for epoxy resin, PU-AN adhesive, and PUR that flax fiber reinforcement reduced the moisture content by 3.76%, 6.7%, and 15%. MUF bonded plywood displayed the highest moisture content of 11.85% and also a high standard deviation of 2.00.

Moisture content (MC) is influenced by the type of adhesive with a *p*-value 0.000 and fiber reinforcement (*p*-value 0.001). The influence of fiber reinforcement on the moisture content was questioned for urea formaldehyde (*p*-value 0.568) and melamine urea formaldehyde (*p*-value 0.731).

Moisture content of wood and wood based products influence several mechanical properties such as MOE, MOR, compression-, and TS within the hygroscopic region [25]. Aydin et al. (2006) displayed the influence of veneer MC on the mechanical properties of UF and MUF bonded poplar and spruce plywood. Increased veneer MC lowered the MOR, SS, and MOE with the positive effect of decreasing formaldehyde emissions [26]. The effect of decreasing equilibrium moisture content is stated by Bal et al. (2015) for the PF adhesive bonded GF reinforced poplar plywood compared to the control group specimens [6]. 

### 3.3. Tensile Strength

The results for ultimate tensile strength f_t_ and maximum tensile force F_max_ were evaluated with an ANOVA including the factors adhesive type and fiber reinforcement. The influence of the adhesive type was slight given the f_t_ with a *p*-value of 0.057 and R^2^ of 0.165. The maximum tensile force F_max_ was independent of the applied type of adhesive (*p*-value 0.303; R^2^ = 0.091). The factor “fiber reinforcement” slightly influenced f_t_ due to a *p*-value of 0.054 with R^2^ 0.106, whereas F_max_ was independent (*p*-value 0.788; R^2^ = 0.009). The interaction between adhesive type and fiber reinforcement was significant (*p* = 0.001) (Table 4).

For epoxy resin bonded plywood with a mean f_t_ of 76.47 (SD = 4.68) N/mm^2^, the flax fiber reinforcement increased by 9.18% with a mean of f_t_ 83.50 (SD = 4.93) N/mm^2^ and 16.14% for the mean f_t_ of 76.47 (SD = 4.68) N/mm^2^ for glass fiber reinforced specimens. Excluding one outlier and comparing the median f_t_ 93.11 (SD = 7.00) N/mm^2^ for UF flax fiber reinforced with median reference sample with 92.94, there was a slight increase of 0.18%.

In contrast to MUF, the isocyanate MDI based prepolymer adhesive and PUR revealed a negative influence of flax fiber reinforcement on tensile strength f_t_. In detail, MUF flax fiber reinforced samples with a mean f_t_ of 79.64 (SD = 7.15) N/mm^2^ reached 91.77% of the reference sample with a mean f_t_ of 86.78 (SD = 9.99) N/mm^2^, displaying a decline of 8.23%. The isocyanate MDI based prepolymer adhesive bonded flax fiber reinforced plywood mean f_t_ declined by 9.62% compared to the reference mean f_t_ of 94.47 (SD = 7.28) N/mm^2^. PUR flax fiber reinforced mean f_t_ of 78.25 (SD = 8.643) N/mm^2^ decreased by 18.03% compared to the reference mean f_t_ of 95.47 (SD = 8.64) N/mm^2^.

The maximum tensile force F_max_ was reached at approximately 20.00 kN for all test groups excluding the epoxy references with a F_max_ of 17.42 kN.

The Pearson correlation between moisture content and tensile strength f_t_ was significant for the PUR flax fiber reinforced specimen (*p*-value 0.01, R^2^ = 1.00). No correlation was found for epoxy (*p*-value 0.645; R^2^ = 0.281), UF (*p*-value 0.850; R^2^ = 0.054), MUF (*p*-value 0.183; R^2^ = 0.910), and the isocyanate MDI based prepolymer adhesive (PU-AN) flax fiber reinforced specimen (*p*-value 0.889; R^2^ = 0.030).

**Figure 4:**
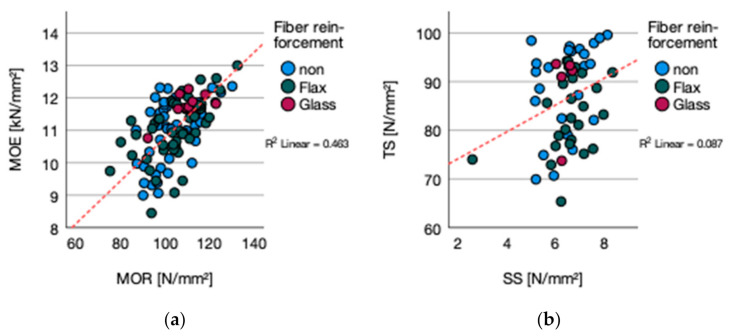
(**a**) Tensile strength vs. density and (**b**) tensile vs. shear strength.

The general stated correlation between increasing density and tensile strength according to Niemz (1993) could not be manifested due to the Pearson correlation with R^2^ = 0.070 and a *p*-value of 0.051 (Figure 4a). Comparing the TS to the range given by Niemz (1993) for plywood between 30 to 60 N/mm^2^, the results exceeded the range by 16.47 (76.47) to 35.46 (95.36) N/mm^2^ [27].

Further research is mandatory for a better understanding of the decline of the tensile strength for MUF, PU-AN, and PUR bonded flax fiber reinforced plywood. The decline could not be explained by bonding performance if compared to the values of the tensile shear strength. Nevertheless, there was a correlation between tensile strength f_t_ and shear strength f_t_ (*p*-value 0.029; R^2^ = 0.087) (Figure 4b). In addition, influences of high moisture content on tensile strength, and maximum tensile strength in the range 5 to 10% MC [27] have to be neglected if compared to the measured moisture content.

### 3.4. Tensile Shear Strength

Shear strength f_t_ (Table 5) was significantly influenced by the type of the adhesive (*p*-value 0.000). Fiber reinforcement had no significant influence on shear strength f_t_ (*p*-value 0.561). Interactions between fiber reinforcement and the applied adhesive type were slight for shear strength f_t_ (*p*-value 0.045).

A general correlation between moisture content, respectively density and shear strength f_t_ was not detected (MC vs. f_t_: R^2^ 0.070; *p*-value 0.697/density vs. f_t_: R^2^ 0.036; *p*-value 0.722).

Comparing the SS means of the applied adhesives, a divergent picture is given (Table 5). Epoxy flax fiber reinforced specimens increased f_t_ by 8.4%, respectively by 3.7% for glass fiber reinforcement in comparison with the mean of the reference samples. UF glued flax fiber reinforced plywood enhanced shear strength ft by 17.0%. MUF bonded flax fiber reinforcement increased by 2.5%. The isocyanate MDI based prepolymer adhesive (PU-AN) bonded flax fiber reinforced plywood mean of 7.20 (SD = 1.96) N/mm^2^ decreased by 5.4% in comparison to the non-reinforced reference mean of 7.61 (SD = 7.61) N/mm^2^. A different picture is seen in the comparison of the median enhancing fiber reinforcement (median 8.07 N/mm^2^) by 6.5%, in contrast to the reference median of 7.58 N/mm^2^. Due to eliminating the influence of the outlier and standard deviation of 1.96, PUR flax fiber reinforced plywood lowered shear strength f_t_ by 5.2%, indicating complications with the glue line.

Bonding strength between veneers is mainly determined by the properties of the adhesives. All specimens exceeded the limit value of 1 N/mm^2^ for the shear strength mean indicated in EN 314-2 [21]. For example, the mean value of UF bonded non fiber reinforced beech plywood with a shear strength of 5.47 N/mm^2^ was three times higher than the findings of Bekhta et al. (2020), with a shear strength mean of 1.51 N/mm^2^ [28]. UF proved to be satisfactory with flax fiber and the adhesive matrix. MUF and PU-AN displayed acceptable improvements. UF is widely used for plywood production due to low price, high bonding strength, and desirable water resistance [29]. The difference between flax and glass fiber for epoxy resin indicates that flax is well suitable. One method to improve the bonding performance between flax fiber and epoxy resin is given by Sbardella et al. (2021), suggesting the use of zinc oxide (ZnO) nanorods [30]. The decrease in PUR indicates adhesive application problems during the manufacturing process. Further testing is mandatory to make a final statement on the suitability of PUR and flax fiber reinforcement. This is due to the fact that polyurethane based adhesives are commonly used for all kinds of applications because of their self-supporting excellent bond strength, fast curing, and environmental influence resistance [31]. In addition, according to Somarathna et al. (2018), several studies have proven the suitability of polyurethane adhesives surpassing the performance of epoxy resins in terms of quasi-static, dynamic, impact, and cyclic loading. Furthermore, the lower costs of polyurethane adhesives compared to epoxy resins [32] should be mentioned. One explanation for the weak bonding performance could be that binding of natural fibers is strongly influenced by their lignocellulosic origin and inherent hydrophilic character, causing weak binding between the fiber and the polymeric adhesive [33]. This is in line with the decreasing post-production moisture content of 15% for PUR compared to the non-reinforced reference, displaying the lowest moisture content value with 8.84% for references and flax fiber reinforced samples. In addition, Lavalette et al. (2016) mentioned an optimum wood moisture content between 30 and 60% for efficient bonding of veneer plies with polyurethane adhesives [34]. To improve the understanding of interaction effects of bonding performance, Li et al. (2020) suggested the combination of lap-shear tests with digital image correlation (DIC) as a valuable investigation method to determine the bonding strength of plywood [29]. 

### 3.5. Modulus of Elasticity and Modulus of Rupture

The MOE and MOR (Table 6) were significantly influenced by the applied type of adhesive (*p*-value 0.00), whereas fiber reinforcement in general had no significant influence on MOE (*p*-value 0.219) and MOR (*p*-value 0.253). Interaction effects between the factors “type of adhesive” and “fiber reinforcement” are given with a *p*-value of 0.00.

Based on the different adhesives, the influence of the factor fiber reinforcement tested by ANOVA varies. For the epoxy resin bonded plywood, no significant influence of fiber reinforcement on MOE (*p*-value 0.198; R^2^ = 0.113) and MOR (*p*-value 0.008; R^2^ = 0.304) could be stated. MOE (*p*-value 0.480; R^2^ = 0.028) and MOR (*p*-value 0.151; R^2^ = 0.111) of the UF bonded plywood was not influenced by the fiber reinforcement. MOE of MUF bonded plywood was slightly influenced by fiber reinforcement (*p*-value 0.003; R^2^ = 0.431) and for MOR (*p*-value 0.107; R^2^ = 0.154), the effect could not be stated. Fiber reinforcement did not affect MOE (*p*-value 0.829; R^2^ = 0.003) and MOR (*p*-value 0.747; R^2^ = 0.006) of the isocyanate MDI based prepolymer adhesive (PU-AN) bonded plywood. Fiber reinforcement significantly influenced MOE (*p*-value 0.000; R^2^ = 0.769) and MOR (*p*-value 0.001; R^2^ = 0.461) of the polyurethane bonded plywood.

Comparing the median for MOE, flax fiber reinforcement increased by 2.6%, respectively by 0.3% for the glass fiber reinforced sample. The UF bonded flax fiber reinforced plywood MOE mean decreased by 2.3%. The MUF bonded flax fiber reinforced specimen increased by 6.5%. The MOE of the isocyanate MDI based prepolymer (PU-AN) bonded flax fiber reinforced plywood increased by 8.9% and the PUR flax fiber reinforced specimen decreased by 11.2%.

The epoxy resin bonded flax fiber reinforced MOR compared by the median increased by 16.65%, respectively by 11.09% for glass fiber reinforcement. The median of MOR for the UF bonded flax fiber reinforced sample lowered by 2.91%. The MUF based flax fiber reinforced plywood increased MOR by 4.65%. The isocyanate MDI based prepolymer flax fiber reinforced specimen improved MOR by 8.20% and for the PUR samples, it decreased by 14.72%.

**Figure 5:**
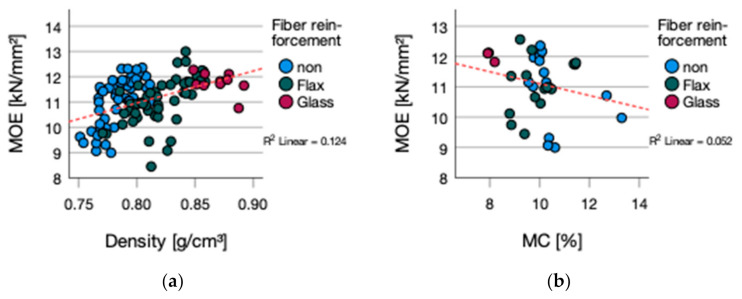
(**a**) MOE vs. density (**b**) MOE vs. MC.

**Figure 6:**
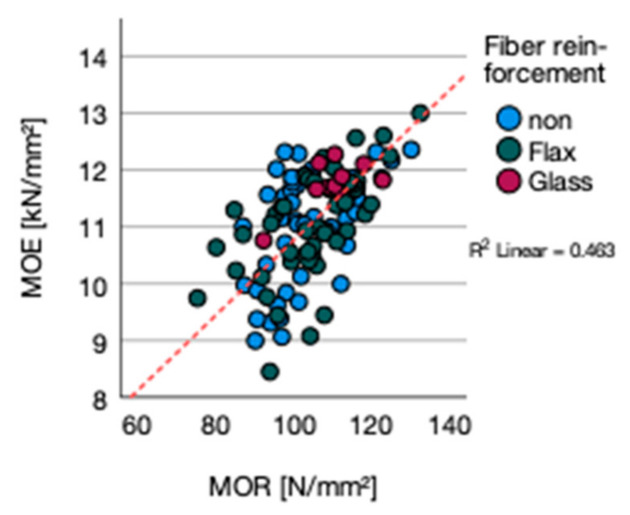
MOE vs. MOR.

The correlation between density and MOE is given (*p*-value 0.000; R^2^ = 0.125) (Figure 5a). A correlation between moisture content and MOE was not detected (*p*-value 0.203; R^2^ = 0.052) (Figure 5b). Within the singular types of adhesives, a correlation for MOE and density was only significant for MUF bonded plywood (*p*-value 0.001; R^2^ = 0.917) and for moisture content (*p*-value = 0.001; R^2^ = 0.854). The general correlation between MOR and MOE is given by a *p*-value 0.000; R^2^ = 0.463 (Figure 6).

MOE is influenced by density, as stated in the literature [25,27]. Flax fiber reinforcement improves MOE, depending on the type of adhesive. The position of the fiber reinforcement within the lay-up strongly influenced the improvements. Fiber reinforcement located closer to the outer layers or at the outside can better contribute to MOE performance due to higher tensile strength within the tension zone [8,14]. In contrast, MOR is strongly dependent on the strength of the surface layer [14]. 

### 3.6. Screw Withdrawal Resistance

**Figure 7:**
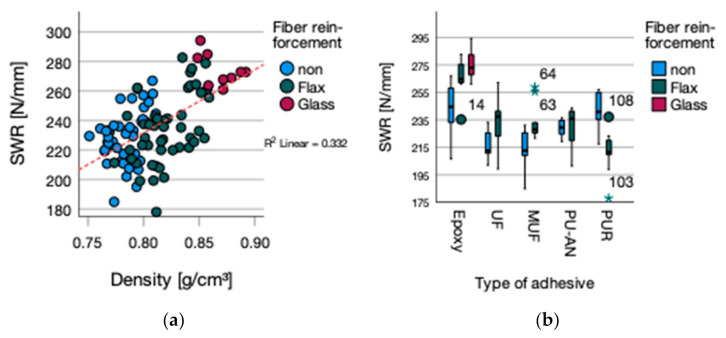
(**a**) SWR vs. density and (**b**) SWR grouped by type of adhesive.

The influence of fiber reinforcement (*p*-value 0.001) and the type of adhesive (*p*-value 0.000) as well as the interaction of both (*p*-value 0.000) are significant. The results for the screw withdrawal resistance display a divergent picture (Figure 7b). Fiber reinforcement improved the screw withdrawal resistance (SWR) median with the exception of PUR bonded flax reinforced plywood (Table 7).

SWR for the epoxy resin flax fiber reinforced mean of 266.28 (SD = 13.98) N/mm increased by 10.13%, respectively by 13.48% for glass fiber reinforced samples, compared to the reference mean of 241.78 (SD = 20.32) N/mm. SWR for the UF bonded flax fiber reinforced mean of 232.79 (SD = 11.21) N/mm improved by 7.02% in contrast to the reference mean of 217.53 (SD = 11.21) N/mm. The MUF glued flax fiber reinforced SWR mean of 233.37 (SD = 14.02) N/mm increased by 9.54% compared to the reference mean of 213.04 (SD = 15.41) N/mm. The isocyanate MDI based prepolymer adhesive (PU AN) bonded flax fiber reinforced SWR sample mean of 228.36 (SD = 15.63) N/mm decreased by 0.34% when comparing the median with an enhancement of 2.66%. The difference between mean and median could be explained by the differences in the SD values for the reference of 6.59 and 15.63 for the reinforced sample. The PUR bonded flax fiber reinforced SWR mean of 211.56 (SD = 14.80) N/mm declined by 12.42% compared to the reference mean of 241.56 (SD = 16.44) N/mm.

The maximum screw withdrawal force Fmax (MSWF) (Table 7) displayed for the epoxy resin bonded flax fiber reinforced plywood was an increase of 20.05% and respectively for glass fiber reinforcement of 15.70%. UF and MUF bonded flax fiber reinforced plywood enhanced by 13.14%, respectively 16.27%. PU AN increased the maximum screw withdrawal force by 7.94%, whereas PUR decreased by 4.49%.

The Pearson correlation (R2 0.332) between density and screw withdrawal resistance (Figure 7a) was significant with a *p*-value of 0.000. This is according to Wagenführ and Scholz (2008), who stated the relation between increasing density and enhanced screw withdrawal resistance [24].

In general, screw withdrawal strength is dependent on screw penetration length, screw diameter, angle between screw and wood fiber direction, wood species, wood moisture content, and temperature. This demonstrates that SWR perpendicular to the wood fiber orientation creates the highest values compared to fiber direction [35]. Furthermore, for laminar wood based composite structures, based on the research of Liu and Guan (2019), the location of fiber reinforcement influences the SWR, suggesting a combination of fiber reinforcement close to the plywood core plies and to the surface plies [36]. This is confirmed by comparing the results of maximum screw withdrawal force F_max_ to Bal et al. (2017). This demonstrates that MSWF improved by 13.65% respectively by 14.11%, if fiber reinforcement is located on the surface within the outer glue lines of the five layered (veneer thickness 2.7 mm) PF bonded poplar plywood reinforced with woven glass fiber fabric (areal weight 500 g/m^2^) [7]. Similar effects have been reported by Kramár et al. (2020). The SWR for basalt fiber reinforcement located at the core layer of particleboards did not enhance the SWR. This was based on the assumption that the degree of compaction in particleboards is lower in the core layer than on the surface, thus affecting the SWR. In addition, this is due to the difference in density between the core (low density) and the surface layer (high density). Fiber reinforcement placed within the surface layer increased the SWR due to increased density caused by a higher degree of compaction within the surface layer [14].

This leads to the conclusion that increasing density is not the singular factor to influence the SWR. Research conducted by Maleki et al. (2017) highlighted that screw withdrawal perpendicular to grain displays a failure mode combination of splitting, caused by tension perpendicular to grain and rolling shear failure [37]. Further aspects such as glue line quality have to be taken into consideration. This is due to the fact that PUR reduced SWR and maximum screw pull-out force Fmax, indicating poor glue line bonding quality. In addition, research should focus on fiber and textile characteristics and screw pull-out behavior within the fiber adhesive matrix and the surrounding veneer plies for a deeper understanding of failure mode and interaction effects regarding SWR.

## 4. Conclusions

**Figure 8:**
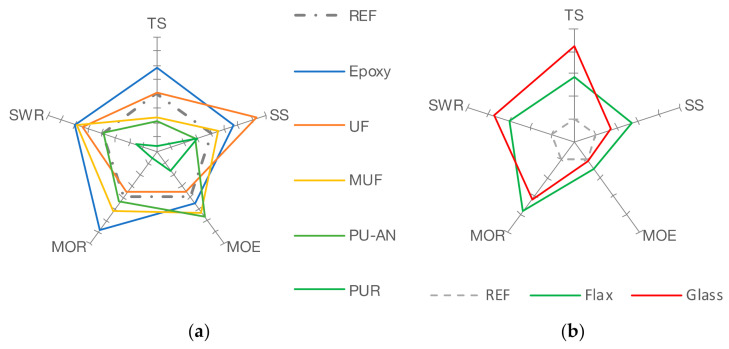
(**a**) Performance of flax fiber reinforcement (axis interval 5%). (**b**) Comparison of the flax and glass fiber reinforcement (axis interval 5%).

Comparing the percentage-based performance (Figure 8a; axis interval 5%) of the different adhesives with flax fiber reinforcement, it can be stated that epoxy resin is well suitable for improving MOE, MOR, TS, SS, and SWR. The UF, MUF, and isocyanate MDI based prepolymer adhesive (PU-AN) increased the performance of the mechanical properties of MOE, MOR, SS, and SWR, but lowered tensile strength compared to the singular references. PUR failed to suit flax fiber reinforcement.

SWR was significantly influenced by the factor flax fiber reinforcement. For MOE, MOR, and SS, no significant influence of flax fiber reinforcement could be stated. TS was slightly influenced by the factor fiber reinforcement.

The results show the possibility of improving mechanical plywood properties by using reinforcing flax fiber fabrics bonded with different industrial standard adhesive systems (Figure 8b; axis interval 5%). Flax fiber reinforcement exceeded the glass fiber reinforced epoxy resin bonded plywood in terms of SS (+5.14%), MOE (+2.3%) and MOR (+3.54%). The SWR for flax reinforced epoxy resin bonded plywood was 3.35% lower and for TS, it was 6.96% lower.

Further research is mandatory to determine the influence of press parameters such as pressure, temperature, and time, in addition to factors like veneer and flax fiber fabric, moisture content or the influence of pre-treatment of the flax fiber fabric to improve bond ability. In addition, research on the fiber reinforcement location within plywood lay-up is necessary in order to optimize the mechanical properties of flax fiber reinforced plywood. Furthermore, research on the influence of formaldehyde emissions caused by UF [38] will have to be conducted. 

## Figures and Tables

**Table 1:** Design of the experiment for the influence of adhesive systems on flax fiber reinforced plywood.

Adhesive	Type of Reinforcement	Adhesive Applic.	Board Thickn.	Density	MC	TS	SS	MOE	MOR	SWR
		(g/m^2^)	(mm)	Number of Specimens (N)						
Epoxy	non	200	10	10	3	5	9	10	10	9
	flax		11.2	10	3	5	9	10	10	9
	glass		11.2	10	3	5	9	10	10	9
UF	non	160	10	10	3	5	9	10	10	9
	flax		11.2	10	3	5	9	10	10	9
MUF	non	300	10	9	3	5	9	10	10	9
	flax		11.2	9	3	5	9	10	10	9
PU-AN	non	200	10	10	3	5	9	10	10	9
	flax		11.2	10	3	5	9	10	10	9
PUR	non	150	10	10	3	5	9	10	10	9
	flax		11.2	10	3	5	9	10	10	9

**Table 2:** Density of different specimens.

Adhesive	Reinforcement	N	Density (g/cm^3^)			
			Min	Mean	Max	SD
Epoxy	non	10	0.79	0.80	0.81	0.01
	flax	10	0.83	0.84	0.86	0.01
	glass	10	0.85	0.87	0.89	0.02
UF	non	10	0.77	0.78	0.79	0.01
	flax	10	0.79	0.81	0.83	0.01
MUF	non	9	0.77	0.79	0.81	0.01
	flax	9	0.82	0.84	0.86	0.01
PU-AN	non	10	0.75	0.77	0.78	0.01
	flax	10	0.80	0.82	0.83	0.01
PUR	non	10	0.77	0.79	0.80	0.01
	flax	10	0.77	0.79	0.81	0.01

**Table 3:** Postproduction moisture content.

Adhesive	Reinforcement	N	Moisture Content (%)	
			Mean	SD
Epoxy	non	5	9.83	0.14
	flax	5	9.46	0.23
	glass	5	8.03	0.15
UF	non	5	10.27	0.09
	flax	5	10.33	0.14
MUF	non	5	11.85	2.00
	flax	5	11.42	0.04
PU-AN	non	5	10.45	0.15
	flax	5	9.75	0.32
PUR	non	5	10.04	0.05
	flax	5	8.84	0.04

**Table 4:** Tensile strength f_t_.

Adhesive	Reinforcement	N	Tensile Strength (N/mm^2^)			
			Min	Mean	Max	SD
Epoxy	non	5	69.94	76.47	82.46	4.68
	flax	5	76.20	83.50	88.71	4.92
	glass	5	73.76	88.81	93.65	8.48
UF	non	5	88.57	93.16	98.44	3.56
	flax	5	78.04	90.51	94.28	7.00
MUF	non	5	70.70	86.78	96.72	10.00
	flax	5	72.93	79.64	90.74	7.15
PU-AN	non	5	82.13	94.47	99.62	7.28
	flax	5	74.02	85.32	91.92	7.37
PUR	non	5	91.66	95.46	97.24	2.20
	flax	5	65.38	78.25	89.56	8.64

**Table 5:** Shear strength.

Adhesive	Reinforcement	N	Shear Strength (N/mm^2^)			
			Min	Mean	Max	SD
Epoxy	non	9	5.20	6.28	6.92	0.58
	flax	9	4.80	6.81	7.70	0.86
	glass	9	6.03	6.51	6.88	0.28
UF	non	9	5.01	5.47	5.83	0.29
	flax	9	5.29	6.40	6.83	0.44
MUF	non	9	5.03	6.29	7.18	0.78
	flax	9	5.82	6.45	7.17	0.50
PU-AN	non	9	6.99	7.61	8.14	0.32
	flax	9	2.59	7.20	8.82	1.96
PUR	non	9	6.49	6.74	7.19	0.23
	flax	9	6.08	6.39	6.84	0.28

**Table 6:** MOE and MOR.

Adhesive	Reinforcement	N	MOE (N/mm^2^)				MOR (N/mm^2^)			
			Min	Mean	Max	SD	Min	Mean	Max	SD
Epoxy	non	10	11,011	11,738	12,099	323	87.00	99.30	109.59	6.41
	flax	10	11,297	12,071	13,000	555	84.84	113.57	137.57	13.24
	glass	10	10,762	11,772	12,277	416	92.31	109.99	122.92	8.04
UF	non	10	9995	11,259	12,317	623	109.63	114.84	121.24	3.05
	flax	10	9448	11,056	11,663	638	106.39	112.15	119.93	4.78
MUF	non	9	9976	10,917	11,700	508	87.43	99.44	109.87	6.63
	flax	9	11,055	11,620	12035	330	94.25	105.58	115.29	8.52
PU-AN	non	10	8997	9530	10,130	366	90.19	95.65	101.92	4.26
	flax	10	3885	9384	10,742	2075	50.31	97.50	111.09	17.33
PUR	non	10	11,427	11,962	12,360	319	97.77	110.87	130.24	12.16
	flax	10	9745	10,477	11,355	517	75.33	91.64	103.76	9.63

**Table 7:** SWR and MSWF.

Adhesive	Reinforcement	N	SWR (N/mm)				MSWF (kN)			
			Min	Mean	Max	SD	Min	Mean	Max	SD
Epoxy	non	9	206.75	241.78	266.98	20.32	2.253	2.643	2.926	0.223
	flax	9	235.33	266.28	282.72	13.98	2.829	3.173	3.378	0.160
	glass	9	261.06	274.36	294.26	10.84	2.937	3.058	3.240	0.104
UF	non	9	202.07	217.53	233.21	11.21	2.186	2.322	2.488	0.113
	flax	9	199.32	232.79	262.20	17.72	2.268	2.627	2.894	0.180
MUF	non	9	184.91	213.04	231.10	15.41	1.993	2.292	2.507	0.181
	flax	9	221.70	233.37	258.95	14.02	2.498	2.665	2.952	0.158
PU-AN	non	9	219.24	229.14	236.55	6.59	2.331	2.444	1.522	0.067
	flax	9	201.40	228.36	243.71	15.63	2.348	2.638	2.807	0.175
PUR	non	9	217.40	241.56	257.15	14.80	2.337	2.605	2.769	0.162
	flax	9	178.00	211.56	237.22	16.44	2.102	2.488	2.802	0.189
